# Molecular Analysis of* CYP21A2* Gene Mutations among Iraqi Patients with Congenital Adrenal Hyperplasia

**DOI:** 10.1155/2016/9040616

**Published:** 2016-09-29

**Authors:** Ruqayah G. Y. Al-Obaidi, Bassam M. S. Al-Musawi, Munib Ahmed K. Al-Zubaidi, Christian Oberkanins, Stefan Németh, Yusra G. Y. Al-Obaidi

**Affiliations:** ^1^Genetic Counseling Clinic and Genetics Laboratory, The Teaching Laboratories, Medical City, Baghdad, Iraq; ^2^Department of Pathology, College of Medicine, Baghdad University, Baghdad, Iraq; ^3^Department of Pediatrics, College of Medicine, Baghdad University and Pediatric Endocrine Consultation Clinic, Children Welfare Hospital, Baghdad, Iraq; ^4^ViennaLab Diagnostics GmbH, Gaudenzdorfer Guertel 43-45, 1120 Vienna, Austria; ^5^National Center of Hematology, Al-Mustansiriya University, Baghdad, Iraq

## Abstract

Congenital adrenal hyperplasia is a group of autosomal recessive disorders. The most frequent one is 21-hydroxylase deficiency. Analyzing* CYP21A2* gene mutations was so far not reported in Iraq. This work aims to analyze the spectrum and frequency of* CYP21A2* mutations among Iraqi CAH patients. Sixty-two children were recruited from the Pediatric Endocrine Consultation Clinic, Children Welfare Teaching Hospital, Baghdad, Iraq, from September 2014 till June 2015. Their ages ranged between one day and 15 years. They presented with salt wasting, simple virilization, or pseudoprecocious puberty. Cytogenetic study was performed for cases with ambiguous genitalia. Molecular analysis of* CYP21A2* gene was done using the CAH StripAssay (ViennaLab Diagnostics) for detection of 11 point mutations and >50% of large gene deletions/conversions. Mutations were found in 42 (67.7%) patients; 31 (50%) patients were homozygotes, 9 (14.5%) were heterozygotes, and 2 (3.2%) were compound heterozygotes with 3 mutations, while 20 (32.3%) patients had none of the tested mutations. The most frequently detected mutations were large gene deletions/conversions found in 12 (19.4%) patients, followed by I2Splice and Q318X in 8 (12.9%) patients each, I172N in 5 (8.1%) patients, and V281L in 4 (6.5%) patients. Del 8 bp, P453S, and R483P were each found in one (1.6%) and complex alleles were found in 2 (3.2%). Four point mutations (P30L, Cluster E6, L307 frameshift, and R356W) were not identified in any patient. In conclusion, gene deletions/conversions and 7 point mutations were recorded in varying proportions, the former being the commonest, generally similar to what was reported in regional countries.

## 1. Introduction

The term congenital adrenal hyperplasia (CAH) comprises a group of autosomal recessive disorders, each due to a deficiency of an enzyme involved in the synthesis of cortisol, aldosterone, or both. The most common form of CAH results from deficiency of the 21-hydroxylase enzyme (aka 21OHD), accounting for about 95% of cases, due to mutations or deletions of* CYP21A2* gene located on 6p21.3 [1].

The condition is usually characterized by either the severe classical form, which includes the salt wasting and simple virilizing forms (manifesting themselves earlier in life), or milder nonclassical or “late-onset” form [2].

21OHD is the most common cause of ambiguous genitalia in female newborns. Affected females are presented with varying degree of genital ambiguity [3]. About 70% of children with classical 21OHD have the salt wasting form, which results primarily from deficient aldosterone synthesis, while nonclassic 21OHD displays symptoms of androgen excess due to mild-to-moderate overproduction of sex hormones that may present at any age [4].

The prevalence of 21OHD as well as its mutation pattern varies among different ethnic populations [5].

The overall worldwide frequency of CAH is estimated to be about 1 per 15,000 live births [6], having higher rates in some Arab countries, for example, 1 : 6400 in Saudi Arabia [7], 1 : 9030 in the United Arab Emirates [8], and 1 : 8000 in the northern part of Palestine [9]. To date there is no report about the incidence or prevalence of CAH among Iraqi people.

Diagnostic challenges arise from similarity of clinical presentations in different enzyme deficiency states causing CAH (21OHD and 11*β*OHD) in addition to false positive results obtained from neonatal screening tests.

Genetic analysis can be helpful to confirm a diagnosis of CAH, in prenatal diagnosis, in detection of carriers in families having a previously affected child, and to offer better treatment options during pregnancy [10].

## 2. Materials and Methods

Sixty-two unrelated patients with a clinical diagnosis of 21OHD registered at the Pediatric Endocrine Consultation Clinic/Children Welfare Hospital in Baghdad, Iraq, were recruited for this study.

Patients were enrolled when they presented with ambiguous genitalia with/without dehydration or pseudoprecocious puberty in females or when they had normal male phenotype with dehydration and/or pseudoprecocious puberty.

To establish the clinical diagnosis of 21OHD, initial baseline investigations were performed including measurements of serum electrolytes and 17-hydroxyprogesterone (17OHP), as well as abdominal and pelvic ultrasonography and bone age determination. Cytogenetic evaluation was performed only for sex determination in those with genital ambiguity. Patients who showed 46XY male patterns were excluded from the study.

DNA was extracted from peripheral blood samples. The DNA was amplified in a multiplex polymerase chain reaction (PCR), followed by hybridization to specific wild and mutant oligonucleotide probes designed to detect the 11 most frequent* CYP21A2* mutations as well as >50% of large gene deletions/conversions using the CAH StripAssay Kit (ViennaLab Diagnostics, Vienna, Austria). Point mutations covered by the CAH StripAssay are P30L/Exon 1 (c.89C>T), I2Splice/Intron 2 (c.290-13A/C>G), Del 8 bp/Exon 3 (c.329_336 delGAGACTAC), I172N/Exon 4 (c.515T>A), Cluster E6/Exon 6 (c.707T>A, c.710T>A, and c.716T>A), V281L/Exon 7 (c.841G>T), L307 frameshift/Exon 7 (c.920-921insT), Q318X/Exon 8 (c.952C>T), R356W/Exon 8 (c.1066C>T), P453S/Exon 10 (c.1357C>T), and R483P/Exon 10 (c.1448G>C). The amplification, hybridization, and detection procedures were performed as reported previously [15].

The study was approved by the ethical committee at the College of Medicine, University of Baghdad, Baghdad, Iraq, and informed consent was obtained from parents of all enrollees.

## 3. Results

Out of 62 unrelated patients, 47 (75.8%) were females and 15 (24.2%) were males, with a female : male ratio of 3.1 : 1. All patients were Arabs and their ages ranged between 1 day and 15 years [mean ± SD = 24.69 ± 41.07 months].

Fifty-two (82%) cases originated from consanguineous marriages. Fifty-seven (91.9%) patients had the classical form of 21OHD [27 (43.5%) of them had the salt wasting “SW” form and 30 (48.4%) cases had the simple virilizing “SV” form], while the milder nonclassic form was seen in 5 (8.1%) patients and developed later during childhood, Table 1.

Mutations were detected in 42 out of the 62 unrelated patients (67.7%): 31 patients were homozygous for one mutation, 9 patients were heterozygotes, 2 patients were compound heterozygotes with 3 different mutations, and the remaining 20 (32.3%) patients harboured none of the tested mutations.

Mutations were subsequently divided into large gene deletions/conversions and point mutations.

Homozygous large gene deletions/conversions were found in 12 (19.3%) patients (Table 2) and as follows:

- Five (8.1%) cases had deletions extending from Cluster E6 to p.R356W.
- Three (4.8%) cases had P30L, I2Splice, and Del 8 bp.
- Two (3.2%) cases had a large deletion/conversion ranging from P30L to I172N.
- Two (3.2%) cases had a complete homozygous gene deletion.

Seven out of the 11 point mutations covered by the CAH StripAssay were detected in the enrolled cases; the remaining 4 mutations (P30L, Cluster E6, L307 frameshift, and R356W) were not detected in the studied cases.

Point mutations were detected in 30 (48.4%) out of the total 62 cases (Tables 3 and 4).

The most frequent mutations were I2Splice and Q318X detected in 10 cases each, followed by I172N detected in 5 cases, V281L detected in 4 cases, Del 8 bp detected in 3 cases, and finally P453S and R483P which were found in one case each.

We subsequently compared genotypes with clinical manifestations; in 27 (43.5%) patients with a classic salt wasting form, we found 22 (81.5%) mutations. The most frequent mutations observed were large deletions/conversions, followed by I2Splice, Q318X, Del 8 bp, and multiple mutations. Five (18.5%) cases showed none of the investigated mutations (Table 5).

Among 30 (48.4%) patients with the simple virilizing form 20 (66.7%) carried a mutation. I172N was the most frequent, followed by Q318X, V281L, I2Splice, deletions/conversions, multiple mutations, P453S, and R483P, while 10 (33.3%) cases showed no mutation.

In 5 (8.1%) cases with nonclassic late-onset form, no mutation was detected (Table 5).

## 4. Discussion

Molecular analysis of common inherited diseases causing major health problems in Iraq was the center of attention of some recent studies [16–19], aiming to build up a database for disease-causing mutations in this region of the world.

As part of this group of diseases, severe forms of CAH can lead to life-threatening conditions if untreated. Genital ambiguity in affected females is known to have a strong impact on social behaviour and health of patients and also represents a significant burden for health care providers. Further complications include the issue of determining the specific enzyme deficiency in some cases.

Patients were recruited from one of the largest pediatric endocrine clinics in Baghdad, Iraq. Yet, the laboratory diagnostic ability is still limited and discrimination of specific enzyme deficiency remains largely based on clinical criteria and follow-up rather than on solid laboratory confirmation.

For this reason, from all the enrolled 62 unrelated Iraqi CAH patients only the children and not their carrier parents were chosen. The* CYP21A2* mutation analysis was done to confirm the clinical suspicion and to study the spectrum as well as frequency of the mutations along with their clinical impact.

Our findings showed that large deletions/conversions of the* CYP21A2* gene were the most common type of mutation among the group of 42 Iraqi CAH patients where a mutation was detected. In addition, 7 out of 11 screened point mutations were detected in several combinations:

- Large deletions/conversions (19.3%) were also described as the most frequent mutation detected in Iran (31.8%) [12].
- The I2Splice mutation (14.5%) was described as the most frequent mutation detected in some regional countries such as Jordan (35.7%) [11], Iran (28%) [13], and Turkey (22%) [10] and in Western Europe [20].
- The Q318X mutation (11.3%) has been described as the most frequent mutation found in Tunisia (35.3% and 26%) [14, 21].
- The V281L mutation was found in 4.0% of the patients. Interestingly in one case a 2-year-old female with ambiguous genitalia and hypertension only carried a heterozygous V281L mutation. Hypertension and genital ambiguity are typically a sign of 11*β*-hydroxylase deficiency. The clinical symptoms in conjunction with a V281L mutation suggest a potential* CYP11B2* mutation causing concomitant 11*β*-hydroxylase deficiency.
- Some alleles carried more than one mutation. In this study, two cases with classic presentation had complex alleles comprising 3 mutations (I2Splice, Del 8 bp, and Q318X). This is similar to findings of Baş et al. who found one SW case with a complex allele comprising R356W, V281L, and I172N [10]. Also Kharrat et al. detected one case homozygous for 2 different mutations (I2Splice + Q318X/I2Splice + Q318X) in Tunisia [14]. A German study reported 5 cases with complex alleles, where 3 patients had I172N-F306+t, one case had I172N-R356W, and one case had V281L-R356W [22].
- P453S was found in a single allele (0.8%), which was also detected by Baş et al. in Turkey but in two alleles [10].
- R483P was uniquely found in a single allele (0.8%) but was not detected in the neighboring countries, Table 6.

Four isolated mutations were tested but not identified in any of our cases, namely, P30L, R356W, Cluster E6, and L307 frameshift mutation.

- The absence of the P30L mutation is similar to the studies performed in Iran, Turkey, and Tunisia [10, 12–14], while it was reported in Jordan [11], Greece [22], Romania [23], Italy [24], and China [25].
- R356W and Cluster E6 were found in Jordan (3.5%, 1.7%) [11] and Iran in 2 studies (7.95%, 2.27%) [12] and (5%, 4%) [13] and in Turkey (8.8%, 2.2%) [10], but not in our cases.
- A single mutation detected in one (0.8%) case in this study, namely, R483P, was not found in any of the neighboring countries [7, 10, 12].
- The L307 frameshift mutation was not detected in this study, as well as in Iran, Turkey, and Saudi Arabia, and was not tested in Jordan.
- All these mutations were detected in the middle European countries [26].

The percentage of alleles with no mutation in this study (39.5%) was high in comparison to other studies [Turkey (15.4%, 22%) [10, 27], Iran (0, 30%) [12, 13], and Tunisia (5.9%)] [14]. Sanger sequencing that has been used in some of the other studies can also detect rare mutations (around 5%) which are not covered by the CAH StripAssay, but it fails to detect larger deletions/conversions. In contrast to Sanger sequencing the StripAssay can detect >50% of large deletions/conversions. However, for the remaining percentage of large aberrations techniques to define the gene copy number need to be applied (MLPA, real-time PCR).

Being an autosomal recessive disorder, 21OHD should affect both sexes equally [28], but the female predominance noted in this study (Table 1) was affected by the type of clinical presentation in this disease causing social problems and impact on health from virilization and dehydration in female patients while males with severe salt loss may die undiagnosed during the neonatal period, whereas moderately affected individuals may stay undetected for several years until symptoms and signs of androgen excess develop [29]. This finding was similarly reported in a Syrian study [30].

Parental consanguinity was found in 51 (82.3%) cases, which reflects the relatively high consanguinity rate among Iraqi population reported to range between 35 and 60% [31]. Similar situations have been seen in other Arab studies (e.g., Saudi Arabia) in which all cases originated from consanguineous marriages.

An interesting observation in the current study is the relatively large number of patients with heterozygous mutations having two related parents [9 (17.7%)]. This finding was similar to studies performed in Tunisia and Turkey, which showed a high frequency of compound heterozygosity (17.6% and 34.8%, resp.) despite a high consanguinity rate [10, 14] which reflects the high frequency and diversity of mutations in these populations, as well as ours.

Among 11 clinical cases with heterozygous mutations, 9 had only a single detectable mutation. We therefore think that the other allele could host a rare and/or novel mutation or a type of large deletion/conversion not covered by the StripAssay. Baş et al. [10] reported similar findings in 10 cases with one mutant allele (using southern blot (SB) and allele-specific semiquantitative PCR/enzyme restriction and sequencing).

Furthermore, the* CYP21A2* promotor region has been reported by several authors to be responsible for contributing to CAH conditions. For instance, in one Chinese study a point mutation in the promoter region of* CYP21A2* gene has been shown to reduce the transcription activity by 80% causing CAH [32].

Thus, we are planning to include Sanger sequencing combined with dosage analysis for both, the 9 cases with a single mutation and the 20 undetermined cases. In-depth analysis might extend the spectrum of mutations currently present in our cohort of patients.

Overall, most cases in this study demonstrated good genotype correlation with their expected phenotype.

In the salt wasting and simple virilizing group, genotype-phenotype correlation was as expected, except for two cases: the first carried a homozygous deletion/conversion—supposedly causing severe enzyme deficiency—but it showed a simple virilizing phenotype. This may be explained by improvement of aldosterone biosynthesis in salt wasting form [33, 34].

The second case had homozygous V281L mutation supposedly causing mild nonclassic form but it presented with simple virilization; this may be due to another undetected alteration in the same allele and should be ruled out by gene sequencing [35].

In conclusion, the current study is the first report about the spectrum and frequency of* CYP21A2* mutations among the Arab CAH patients of Iraq showing a high frequency and diversity. Large deletions/conversions were most frequent. Seven out of 11 tested point mutations were also reported, I2Splice and Q318X being the commonest. R483P was uniquely identified in one case only. Female predominance and parental consanguinity were reported at a higher frequency than the general population with a generally good genotype/phenotype correlation.

## Figures and Tables

**Table 1:** Clinical presentation and age distribution of Iraqi CAH patients with 21-hydroxylase enzyme deficiency.

Clinical presentation	Females number (%)	Males number (%)	Total number (%)	Age range (mean ± SD)
Classic				
Salt wasting form	20 (32.3%)	7 (11.3%)	27 (43.6%)	1 day–3 months (1.01 ± 1.37) months
Simple virilizing form	23 (37.1%)	7 (11.3%)	30 (48.4%)	1 day–14 years (2.61 ± 3.16) years
Nonclassic form (pseudoprecocious puberty)	4 (6.4%)	1 (1.6%)	5 (8%)	8–15 years (9.4 ± 3.13) years

Total	47 (75.8%)	15 (24.2%)	62 (100%)	1 day–15 years (24.69 ± 41.07) months

**Table 2:** Distribution of Iraqi CAH cases with 21-hydroxylase enzyme deficiency with large deletions/conversions of *CYP21A2* gene.

Types of deletions/conversions	Number (%)
P30L, I2Splice, Del 8 bp	3 (4.8)
P30L, I2Splice, Del 8 bp, I172N	2 (3.2)
Cluster E6, V281L, L307 frameshift, Q318X, R356W	5 (8.1)
Complete *CYP21A2* gene deletion	2 (3.2)

*Total*	*12 (19.3)*

**Table 3:** Distribution of mutations in the tested alleles and zygosity status among Iraqi CAH cases.

	Homozygotes number (%)	Heterozygotes number (%)	Compound heterozygotes number (%)	Total detected number (%)	Undetected number (%)	Total number (%)
Cases	31 (50) (i) 12 Del (ii) 19 PM	9 (14.51) (i) 0 Del (ii) 9 PM	2 (3.23) (i) 0 Del (ii) 2 PM	42 (67.75) (i) 12 (ii) 30	20 (32.25)	*62 (100)*
Tested alleles	62 (50)	18 (14.51) (i) 9 detected (7.25) (ii) 9 undetected (7.25)	4 (32.23)	75 (60.48)	49 (39.52)^*∗*^	*124 (100)*
Mutations	62 (50) (i) 24 Del (ii) 38 PM	9 (7.25) (i) 0 Del (ii) 9 detected PM (iii) 9 undetected^*∗*^	6 (4.83) (i) 0 Del (ii) v6 PM^*∗∗*^	77 (62.1)^#^ (i) 24 Del (ii) 53 PM	49 (39.52)	*124 expected mutations* ^Ψ^

Del = large deletion/conversion. PM = point mutation.

^*∗*^40 alleles from 20 homozygous cases plus 9 heterozygous cases where only one mutation was found.

^*∗∗*^Those two cases each carry 3 point mutations in their 2 alleles (I2Splice, Q318X, and Del 8 bp).

^#^77 mutations were detected in 75 alleles.

^Ψ^126 total number of mutations (77 were detected and 49 undetected) instead of 124 expected mutations.

**Table 4:** Frequency of detected *CYP21A2* mutations and zygosity status among Iraqi CAH patients.

Type of mutation	Number of cases (number of alleles carrying that mutation)		Number (%) of mutant alleles
	Homozygous	Heterozygous	
P30L	0	0	*0*
I2Splice	8 (16)	2^*∗*^ (2)	18 (14.5%)
Del 8 bp	1 (2)	2^*∗*^ (2)	4 (3.2%)
I172N	5 (10)	0	10 (8.1%)
Cluster E6	0	0	0
V281l	1 (2)	3 (3)	5 (4.03%)
L307 frameshift	0	0	0
Q318X	4 (8)	4 + 2^*∗*^ (6)	14 (11.3%)
R356W	0	0	0
P453S	0	1 (1)	1 (0.81%)
R483P	0	1 (1)	1 (0.81%)
Large deletions/conversions	12 (24)	0	24 (19.3%)

*Total detected*	*31 (62)*	*11 (13)* ^$^	77 mutations in 75 alleles (60.5%)

*Undetected*	*20 (40 + 9)* ^#^		49 (39.5%)

*Total cases*	*62 (124)*		

^*∗*^Two of these heterozygous cases were compound, each having 3 mutations (namely, I2Splice, Del 8 bp, and Q318X) in their 2 alleles.

^#^Additional 9 alleles were undetected in the 9 heterozygous cases; only one mutation was detected.

^$^Thirteen mutant alleles were detected in 11 patients (nine of them had a single detected mutation but the remaining two had 3 mutations as mentioned above^*∗*^).

**Table 5:** Frequency of *CYP21A2* mutations according to clinical presentation in 62 Iraqi CAH patients.

Mutation type	Number of patients (%)			
	SW	SV	NC	Total
Large deletions/conversions	11 (40.7)	1 (3.3)	0 (0.0)	12 (19.3)
Q318X	4 (14.8)	4 (13.3)	0 (0.0)	8 (12.9)
I2Splice	5 (18.5)	3 (10.0)	0 (0.0)	8 (12.9)
I172N	0 (0.0)	5 (16.7)	0 (0.0)	5 (8.1)
V281L	0 (0.0)	4 (13.3)	0 (0.0)	4 (6.4)
Del 8 bp	1 (3.7)	0 (0.0)	0 (0.0)	1 (1.6)
Multiple mutations (I2Splice, Del 8 bp, Q318X)	1 (3.7)	1 (3.3)	0 (0.0)	2 (3.2)
P453S	0 (0.0)	1 (3.3)	0 (0.0)	1 (1.6)
R483P	0 (0.0)	1 (3.3)	0 (0.0)	1 (1.6)

*Total detected mutations*	*22 (81.5)*	*20 (66.7)*	*0 (0.0)*	*42 (67.7)*

Not detected	5 (18.5)	10 (33.3)	5 (100)	20 (32.3)

*Total*	*27 (100)*	*30 (100)*	*5 (100)*	*62 (100)*

**Table 6:** Frequency of common allelic mutations in this study compared with previous studies from some of the neighboring and Arab countries.

	Present study, Iraq, 2015	Turkey, 2009 [10]	Jordan, 2011 [11]	Iran, 2011 [12]	Iran, 2008 [13]	Tunisia, 2004 [14]
Total alleles (*n*)	124	91	92	88	100	102
Not detected (%)	39.5%	15.4%	40%	0	30%	5.9%
Molecular method(s) used	AS-PCR with reverse hybridization for 11 mutations	SB, AS-PCR/ER, and sequencing	ARMS for 8 common mutations & MLPA	AS-PCR for 8 common mutations and sequencing	AS-PCR for 8 mutations	ER + sequencing
P30L	0	0	17.8	0	0	0
I2Splice	14.5	22.0	35.7	14.77	28	17.6
Del 8 bp E3	3.2	4.4	16	0	13	0
I172N	8.1	9.9	16	5.68	9.0	10.8
Cluster E6	0	2.2	1.7	2.27	4.0	0
V281L	4.0	7.6	0	1.14	3.0	0
L307fs	0	0	NT	0	NT	0
Q318X	11.3	3.3	23.2	15.91	9	35.3
R356W	0	8.8	3.5	7.95	5.0	2.0
P453S	0.81	2.2	NT	NR	NT	NR
R483P	0.81	NR	NT	NR	NT	
Deletions/conversions	19.3	8.8/14.3	0	31.8	NT	19.6

NR: not reported; NT: not tested; SB: southern blot; AS-PCR: allele-specific oligonucleotide hybridization by PCR; ER: enzyme restriction; ARMS: amplification refractory mutational screen; MLPA: multiplex ligation dependent probe amplification.
